# Expression of miR-15b-5p and toll-like receptor4 as potential novel diagnostic biomarkers for hepatitis C virus-induced hepatocellular carcinoma

**DOI:** 10.1016/j.ncrna.2024.12.003

**Published:** 2024-12-15

**Authors:** Amal Ahmed Mohamed, Noha Nagah Amer, Noha Osama, Wael Hafez, Ali Elsaid Abdelrahman Ali, Mahmoud Maamoun Shaheen, Ayman Abd Alhady Alkhalegy, Eman Alsayed Abouahmed, Shamel Mohamed Soaida, Lamees A. Samy, Ahmed El-Kassas, Ivan Cherrez-Ojeda, Rehab R El-Awady

**Affiliations:** a Department of Biochemistry and Molecular Biology, National Hepatology and Tropical Medicine Research Institute, GOTHI, Cairo, Egypt; b Department of Biochemistry and Molecular Biology, Faculty of Pharmacy (Girls), Al-Azhar University, Cairo, Egypt; c Pediatritic Nutrition, Fitoverfat Nutrition Clinic, Cairo, Egypt; d Internal Medicine Department, Medical Research and Clinical Studies Institute, The National Research Centre, Cairo, Egypt; e Department of Diagnostic and Interventional Radiology, National Hepatology and Tropical Medicine Research Institute, GOTHI, Cairo, Egypt; f Department of Internal Medicine, Faculty of Medicine Cairo University, Cairo, Egypt; g Department of General Surgery, El Sahel Teaching Hospital, GOTHI, Cairo, Egypt; h Department of Clinical Pathology, Minia University, Minia, Egypt; i Department of Clinical Oncology, Faculty of Medicine, Cairo, Egypt; j Department of Clinical Pathology, Cairo University, Cairo, Egypt; k Department of Radiology, Elsahel Teaching Hospital, GOTHI, Cairo, Egypt; l Department of Allergy and Immunology, Universidad Espiritu Santo, Samborondon, Ecuador; m Respiralab Research Group, Guayaquil, Ecuador

**Keywords:** miRNA-15b-5p, Toll-like receptor 4, HCC, HCV, Biomarkers, Diagnostic performance

## Abstract

**Objectives:**

Globally, hepatocellular Carcinoma (HCC) ranks seventh in women's cancer and fifth in men's cancer. Early identification can minimize mortality and morbidity. MicroRNAs and Toll-like receptors have been suggested as potential new biomarkers for HCC; Therefore, we explored Toll-like receptor 4 (TLR-4) and miRNA 15b-5p as new non-invasive HCC biomarkers and early detection approaches.

**Methodology:**

In this case-control study, four primary groups were formed from 400 patients who participated in this study: 100 hepatitis C (HCV) patients without cirrhosis or HCC, 100 HCV with cirrhosis patients, 100 HCC and HCV patients, and 100 healthy controls. The HCC diagnosis was confirmed according to the American Association for the Study of Liver Disease (AASLD) Practice Guidelines. Triphasic computed tomography was used to assess the HCC tumor size. Real-time PCR was used to analyze miRNA 15b-5p and Toll-like receptor 4 (TLR-4) expression profiles.

**Results:**

Significant diagnostic performance was achieved by miRNA 15b-5p in differentiating the HCC group from the control group, with 90 % sensitivity and 88 % specificity (AUC] 0.935, p < 0.001), while TLR-4 had moderate diagnostic performance with 85 % sensitivity and 86 % specificity (AUC:0.885, p < 0.001).

**Conclusions:**

The ability of miR-15b-5p to recognize HCC was positive and it outperformed Toll-like receptor4. MiR-15b-5p has the potential to be a more precise and predictive biological marker for HCC than Toll-like receptor4. Future studies exploring different miRNAs and HCC cases from various etiologies are required to better understand the role of miRNAs in this disease and allow for more effective strategies.

## 1 Introduction

With the highest incidence and mortality in East Asia and Africa, liver malignancy is the sixth most frequently diagnosed cancer worldwide, and the fourth most common cause of malignancy-related fatalities. Globally, hepatocellular carcinoma (HCC) ranks seventh in women with malignancy and fifth in men with malignancy.

Owing to the different frequencies of associated etiologies, the incidence of HCC varies worldwide; for example, 72 % of cases occur in Asia, with more than 50 % of cases occurring solely in China, while the incidence in North America is 5 % [1]. Mongolia has the highest age-standardized incidence rate (ASIR per 100,000 individuals) and age-standarized mortality rate (ASMR) rates for HCC in East Asia. Remarkably, ASIR and ASMR roughly equalize globally, indicating a high death rate associated with HCC [2]. Egypt ranks third and 15th among the most populated countries in Africa and the world, respectively. It is the fourth most common cancer [3].

A curative strategy requires early detection of HCC, given that tumor cure is only possible when HCC is small. Consequently, patients who are at risk of early diagnosis of small tumors should be screened for HCC [4]. It is evident that noninvasive, measurable biomarkers that can detect HCC early are necessary to lower morbidity and mortality from the disease and to enable the use of more effective and economical surveillance techniques [4].

A biomarker that is optimal for the early diagnosis of HCC should meet specific requirements, including being unique to HCC, requiring little invasiveness to detect, being easy to process, economical, and outperforming existing HCC biomarkers. A good detection performance is required, as measured by the sensitivity, specificity, and area under the receiver operator curve (AUC). Additionally, consistent results across sexes, ethnic groups, and underlying liver disorders are also required. A wide range of new biomarkers in the blood has been the subject of numerous studies to assess their potential for early detection and prediction of HCC. Nonetheless, very few studies have been sufficiently accurate for international groups to recommend their use [5].

Current research has focused on novel approaches to cancer detection, such as those that rely on the detection of microRNAs (miRNAs). MiRNAs are naturally occurring, non-coding, single-stranded RNA molecules that are typically 22 nucleotides in length (nt). As antisense RNA, short RNA molecules regulate the post-transcriptional levels of their target genes. Although the majority of miRNAs very slightly affect the ability of target genes to translate, they nonetheless form extremely intricate networks that include both targets and downstream effectors [6].

Numerous studies have suggested that miR-15b-5p serves as a biomarker for HCC [7]. Numerous malignancies and non-malignant conditions, including metabolic disorders, neuropsychiatric diseases, and cardiovascular illnesses, are affected by this miRNA. Furthermore, it has been reported to exert tumor-suppressive or oncogenic effects in a variety of malignancies [8].

Toll-like receptors (TLRs) mediate inflammatory responses, preserve the integrity of the epithelial barrier, and stimulate several signaling pathways during cancer chemotherapy. The immunomodulatory role of TLRs in cancer genesis, pathology, and treatment approaches has been the subject of research for many years. Nevertheless, new findings indicate that TLRs can signal or cause regulated cell death (RCD), which is crucial to the anticancer process, in addition to initiating innate and adaptive immune responses [9].

One extracellular pathogen recognition receptor (PRR) is toll-like receptor 4 (TLR-4). It can trigger intracellular signaling, which in turn activates transcription factors involved in the transcription of genes relevant to the immune system or cancer. Therefore, TLR-4 may be involved in the pathophysiology of HCC [10].

HCC has a multitude of etiological risk factors, some of which are strongly associated with development. The development of HCC is closely linked to hepatotropic viruses, including hepatitis B virus (HBV), hepatitis C virus (HCV), and hepatitis D virus (HDV). Approximately 80–90 % of HCC cases occur in patients with preexisting cirrhosis [11]. As the majority of HCV-associated HCCs develop in cirrhotic livers, cirrhosis is a major contributing factor to the development of HCCs. Furthermore, even years after reaching systemic vascular resistance (SVR), patients with established cirrhosis continue to have an increased risk of HCC [12].

Chronic liver disorders account for approximately 90 % of all HCC cases. HCC is most strongly associated with cirrhosis of any etiology. With a 1–6% yearly incidence, HCC is the primary cause of death in patients with cirrhosis. Diabetes, nonalcoholic steato-hepatitis (NASH) associated with obesity, persistent alcohol use, and HBV or HCV infection are the main risk factors for HCC. Other less common risk factors include hemochromatosis, α1-antitrypsin deficiency, and cirrhosis triggered by primary biliary cholangitis. In particular, patients with hemochromatosis-related cirrhosis are especially vulnerable to HCC; during their lifetime, up to 45 % of them may acquire HCC [13].

Given the high prevalence in Egypt and the increased risk factors for HCC, accurate and rapid biomarkers are essential for the early detection of HCC, thus reducing its associated morbidity and mortality. Therefore, our study aimed to assess the potential role of miR-15b-5p and Toll-like receptor 4 as biomarkers for the diagnosis of HCC in the sera of Egyptian patients with HCV and/or cirrhosis.

## 2 Patients and methods

### 2.1 Study population

This study was conducted in accordance with the Declaration of Helsinki. IRB approval was obtained from the Faculty of Pharmacy (Girls), Al-Azhar University Ethics Committees (REC No. 364, Approval number 31/2023). A total of 400 participants enrolled from Al-Azhar University Hospital, Cairo, Egypt were included in this case-control study.

The 400 participants were divided into the following groups:100 patients with hepatitis C virus (HCV), 100 patients with HCC, 100 patients with cirrhosis, and 100 healthy controls. Enrollment in this study was conditional on meeting certain screening criteria. These included being male or female, between 23 and 80 years of age at the time of randomization, laboratory and clinical evidence of chronic hepatitis C virus identified using ELISA and HCV-RNA viremia identified by real-time PCR, abdominal ultrasound showing focal hepatic lesions suggestive of malignancy, histologic evaluation confirmed to be HCC and tumor size evaluation using triphasic computed tomography (CT).

The following factors led to patients' disqualification from this study: history of extreme, potentially fatal, or other serious drug sensitivity; use of any herbal supplements within the 2-week period before the first dose of the study drug; pregnant women; or those receiving immunomodulatory interferon-α therapy. Additionally, further exclusion was applied to any hepatic conditions other than chronic HCV infection, encompassing, but not restricted to, the following causes: autoimmune hepatitis, alcoholic liver disease, Wilson's disease, non-alcoholic steatohepatitis, drug-related liver disease, and parasitological, serological, or ultrasonographic results pointing to alternative causes of chronic liver illness, including biliary diseases, malignancies, schistosoma infections, hepatitis B, or simultaneous B and C viral infections.

### 2.2 Specimen collection

Trained laboratory technicians collected fasting venous blood samples (10 ml). Three milliliters was placed in a dry tube to detect serum alanine aminotransferase (ALT), aspartate aminotransferase (AST), total bilirubin, and albumin levels. Two milliliters were collected in sodium citrate tubes to determine the international normalization ratio (INR). A final volume of 5 ml was used for miRNA extraction and detection. All biochemical parameters were measured using a CX4 Bechman biochemistry autoanalyzer. The patients underwent a complete medical history recording, comprehensive clinical examination, and evaluation by laboratory testing, which included liver function tests, schistosomiasis serological diagnosis, viral hepatitis PCR, urine and stool analysis (all routine tests for all patients on admission), and ultrasonography.

### 2.3 Test methods

#### 2.3.1 Determination of miR-15b-5p expression profile

##### 2.3.1.1 RNA isolation & cDNA synthesis

The miRNeasy Mini Kit (Qiagen, Valencia, CA, USA) was used to isolate RNA, which combines phenol/guanidine-based lysis of samples with silica-membrane-based purification of total RNA. QIAzol Lysis Reagent obtained from Qiagen (Valencia, CA, USA, no. 79306) is a monophasic phenol and guanidine thiocyanate solution intended to promote tissue lysis, block RNase activity, and eliminate the majority of cellular DNA and proteins from lysates through organic extraction. RNA can be easily and quickly purified using silica membrane filters, producing pure RNA that is suitable for most applications. Mature miRNAs were identified using TaqMan® miRNA assays, which were performed in compliance with the manufacturer's instructions, utilizing a stem-looped primer for reverse transcription and sequence-specific TaqMan assay.

###### 2.3.1.1.1 Reverse transcription- polymerase chain reaction

Reverse transcription (RT) was used to convert miRNAs into cDNA. Total miRNA was extracted using miRNA-specific stem-loop RT primers (Applied Biosystems, Cat. No.4366596) from TaqMan® miRNA assays, and reagents from the TaqMan® MicroRNA Reverse Transcription Kit (Applied Biosystems, Cat. No. 4427975).

##### 2.3.1.2 Quantitative real-time PCR

TaqMan Gene Expression (Applied Biosystems, Foster City, California, USA) was used to quantify the microRNAs. Every miRNA in this study was compared with RNAU6, which served as a housekeeping gene (endogenous reference cDNA). The starting concentration of the target sequence was expressed as fractional threshold cycles (CT). We used the following arithmetic formula to calculate relative mRNA quantification: 2)^−ΔΔCT^ (^Δ^CT consists of the distinction between a target cDNA's CT and an endogenous reference cDNA). Consequently, this number provides the target amount normalized to an endogenous reference.

#### 2.3.2 quantification of toll-like receptor4

Toll-like receptor-4 gene expression was determined by real-time PCR using TaqMan Gene Expression (Applied Biosystems) after total RNA extraction using a RNesay Mini Kit (Qiagen Inc., CA Cat-No. 74104).

### 2.4 Statistical analysis

IBM Statistical Package for Social Sciences SPSS advanced statistics was used to analyze the data [version 24 *(SPSS* IBM *Inc., Chicago, IL)*].

While the numbers and percentages represent qualitative data, the mean and standard deviation describe the numerical data.

Normality was tested using the Kolmogorov-Smirnov and Shapiro-Wilk tests. An independent *t*-test or Mann-Whitney *U* test was performed. Comparisons between more than two groups were performed using ANOVA or the Kruskal-Wallis test, as appropriate, and pair-wise comparisons were performed using the post-hoc test. The p-values were adjusted for inflation using the Bonferroni correction.

To calculate the sensitivity, specificity, positive predictive value, negative predictive value, and overall accuracy, a receiver operating characteristic (ROC) curve was employed.

## 3 Results

A total of 400 participants were included in the study and categorized into four primary groups: 100 patients with HCV without cirrhosis or HCC, 100 patients with HCV with HCC, 100 patients with cirrhosis, and 100 age- and sex-matched healthy controls.

Table 1 presents the demographic characteristics of the three study groups. The study groups did not show statistically significant differences in age, sex, BMI, or smoking status.

**Table 1:** Comparison between study groups regarding demographic characteristics.

Variables	Control (n = 100)	HCV (n = 100)	Cirrhosis (n = 100)	HCC (n = 100)	P value
Age	53.8 ± 14.7	52.8 ± 10.8	55.4 ± 8.5	53.7 ± 8.1	0.389
Male gender n%	67 (67.0 %)	65 (65.0 %)	65 (65.0 %)	68 (68.0 %)	0.669
BMI	29.3 ± 4.3	29.6 ± 5.7	28.9 ± 2.8	29.4 ± 3.5	0.620
Smoking n%	17 (17.0 %)	15 (15.0 %)	21 (21.0 %)	18 (18.0 %)	0.733

BMI: Body mass index.

The routine biochemical tests for the four groups are shown in Table 2. AST, Creatinine, and INR levels were significantly elevated in the HCC group relative to those in the HCV, Cirrhosis, and control groups. Additionally, a slight increase in these markers was found in the Cirrhosis and HCV groups compared with that in the controls. However, the ALT level was significantly higher in the HCV, HCC, and cirrhosis groups than in the control group. Conversely, ALT levels were slightly lower in the HCC and cirrhosis groups than in the HCV group. Serum bilirubin levels were higher in the cirrhosis group than those in the HCC, HCV, and control groups. In addition, a slight increase in bilirubin level was found in both the HCC and HCV groups compared to that in the controls. Conversely, serum albumin levels were slightly lower in the HCV group than in the control group, and significantly lower in the HCC and cirrhosis groups than in both the HCV and control groups.

**Table 2:** Comparison between study groups regarding laboratory findings.

Variables	Control (n = 100)	HCV (n = 100)	Cirrhosis (n = 100)	HCC (n = 100)	p value
Mean SD ±					
AST (U/L)	28.87 ± 5.36	33.10 ± 7.05^a^	69.97 ± 39.85^a^^,^^b^	104.97 ± 53.85^a^^,^^b^	0.001∗
ALT (U/L)	30.29 ± 5.60	75.45 ± 49.93^a^	46.01 ± 26.19^a^^,^^b^	60.43 ± 28.18^a^^,^^b^	0.001∗
Creatinine (mg/dl)	0.96 ± 0.16	0.97 ± 0.16^a^	1.52 ± 1.14^a^^,^^b^	1.98 ± 0.81^a^^,^^b^	0.001∗
Bilirubin (mg/dl)	0.77 ± 0.19	0.92 ± 0.20^a^	3.68 ± 4.90^a^^,^^b^	2.91 ± 1.79^a^^,^^b^	0.001∗
Albumin (g/dlL)	3.84 ± 0.21	3.77 ± 0.34^a^	2.62 ± 0.44^a^^,^^b^	2.84 ± 0.41^a^^,^^b^	0.001∗
INR	0.98 ± 0.06	1.03 ± 0.10^a^	1.33 ± 0.21^a^^,^^b^	1.43 ± 0.22^a^^,^^b^	0.001∗

AST: Aspartate aminotransferase.

ALT: Alanine transaminase.

INR: International normalized ratio.

(a) Statistically significant difference compared with the control group (p < 0.05).

(b) Statistically significant difference compared with the HCV group (p < 0.05).

**Fig 1:**
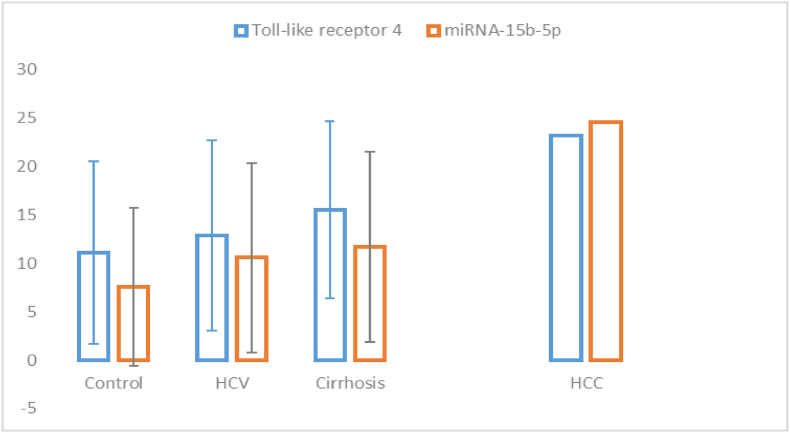
Toll-like receptor 4 and miRNA-15b-5p expression -displayed in error Bars -in different study groups.

Toll-like receptor 4 and miRNA-15b-5p gene expression levels were significantly higher in the HCC group than in the HCV, cirrhosis, and control groups (Table 3, Fig. 1).

**Table 3:** Comparison between studied groups regarding Toll-like receptor 4 and miRNA-15b-5p expression.

Variables	Control (n = 100)	HCV (n = 100)	Cirrhosis (n = 100)	HCC (n = 100)	p-value
	(mean ± SD)	(mean ± SD)	(mean ± SD)	(mean ± SD)	
**Toll-like receptor 4**	11.13 ± 9.420	12.90 ± 9.818(a)	15.48 ± 9.111^(^a^,^^b^^)^	23.16 ± 6.573^(^a^,^^b^^)^	0.001∗
**miRNA-15b-5p**	7.56 ± 8.150	10.56 ± 9.792(a)	11.66 ± 9.840^(^a^,^^b^^)^	24.51 ± 3.806^(^a^.^^b^^)^	0.001∗

(a) Statistically significant difference compared with the control group (p < 0.05).

(b) Statistically significant difference compared with the HCV group (p < 0.05).

**Fig 2:**
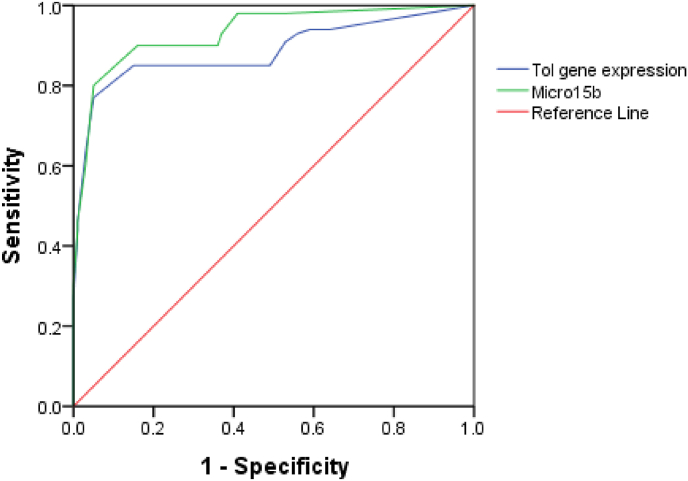
ROC curve for miR-15b-5p and Toll-Like receptor 4 in differentiating the HCC group from the HCV group.

Diagnostic performance was also assessed. Higher diagnostic performance was shown by miRNA-15b-5p in differentiating HCC versus HCV groups, with 90 % sensitivity and 84 % specificity (AUC 0.932, p < 0.001). In contrast, Toll-like receptor 4 demonstrated significantly lower diagnostic performance in the differentiation of HCC versus HCV groups, showing %85 sensitivity and 85 % specificity (AUC:0.885, p < 0.00) (Table 4, Fig. 2).

**Table 4:** Receiver Operating Characteristic curve (ROC) for diagnostic performance and cut points of the miR-15b-5p and Toll-Like receptor 4 in differentiating the HCC group from the HCV group.

Markers	Cut points	Sensitivity	Specificity	AUC	p-value
miR-15b-5p	22.50	90 %	84 %	0.932	0.001^a^
Toll-Like receptor 4	22.70	85 %	85 %	0.885	0.001^a^

AUC: Area under the curve.

a Statistically significant difference at p < 0.05.

## 4 Discussion

HCV, a hepatotropic RNA virus, is a major contributor to chronic liver diseases. Significant morbidity and mortality rates are related to HCC, a major consequence of HCV infection [14].

Prolonged HCV infection is recognized as the fundamental cause of HCC and is a contributing factor for cirrhosis and chronic liver disease (CLD) [15]. The interaction of the immune system, viruses, and overlapping external impacts of host metabolic liver disorders over the last decade suggests that the processes of HCC genesis, progression, and metastasis are exceedingly complex. The inability to easily obtain a model system for HCV infection in animals poses a substantial challenge to comprehending the processes involved in viral carcinogenesis [16].

The expression of miRNAs varies with the development of several cancers, including hepatic cancer, indicating that miRNAs may serve as tumor suppressor genes or new oncogenes in the process of carcinogenesis [17].

Immune responses, including the ability of the extracellular matrix to identify pathogens, depend heavily on TLRs, which are receptors that have been conserved over time from the PRR family. To eradicate pathogenic microorganisms and cancer remnants, TLRs directly regulate inflammatory reactions and initiate immunological responses that are either innate or adaptive [18]. The ability of TLRs to recognize pathogen-associated molecular patterns (PAMPs), conserved pathogen structures, or the damage that pathogens inflict inside the host makes them a crucial component of the first line of protection against infections [19].

The current study included patients with HCC; 68 % of them were males and 32 % were females which agrees with the results of Liu et al. [20], who demonstrated a noticeable male prevalence of HCC. Depending on the demographics, the male-to-female incidence ratio of HCC ranged from 2:1 to 4:1. Compared to women, men are more prone to have established risk factors, including drinking alcohol or smoking cigarettes and persistent HBV or HCV infection, which could account for their greater risk of HCC. Furthermore, inherent risks that reflect a variation in the distribution across genders exist.

In this study, the mean age at the time of HCC diagnosis was 58 years. Our study was aligned with that of Mcglynn et al. [21], who found that the average age was 58 years. In the US, for example, men typically receive diagnoses between the ages of 60 and 64, while women are diagnosed between 65 and 69 years of age. Nonetheless, in Africa, the median age at diagnosis in Egypt (58 years) and other African countries is significantly different (46 years) [22].

Our laboratory work demonstrated that AST, ALT, Bilirubin, Creatinine, and INR were amplified in the HCC, Cirrhosis, and HCV groups compared with controls. Albumin levels were lowest in the HCC group and highest in the HCV group. However [23], it was found by Mourad et al. found that all groups (HCC and HCV) had elevated ALT and AST levels and, in contrast, increased albumin levels. Núñez et al. [24] discovered that a risk factor for early stage tumor growth is hypoalbuminemia (<3.4 g/dL). Carr et al. [25] found that reduced blood albumin levels served as a marker of systemic inflammation and were also linked to elevated parameter measurements of HCC aggression. Subsequently, the aggressiveness of HCC may be influenced by decreased blood albumin levels.

In this study, miRNA-15b-5p performed well as a diagnostic instrument to differentiate the HCC group from the control group, showing 90 % sensitivity and 88 % specificity (AUC 0.935) (p < 0.001). In a study by Pan et al. [26], miR-15b-5p expression levels in HCC were primarily overexpressed compared to non-HCC tissue samples (SMD = 0.618, P < 0.0001), according to a comparison of 456 nearby non-HCC and 991 HCC tissue samples. MiR-15b-5p in HCC demonstrated pooled sensitivity and specificity of 72 % and 68 %, respectively (SROC = 0.81).

In contrast, Yi et al. [27] found that miR-15b-5p expression in patients with HCC had an AUC of 0.485 (98.25 % sensitivity and 15.25 % specificity). According to another study [7], the miR-15b-5p AUC value for HCC identification differed across research groups (HCC compared to cirrhosis, healthy subjects, or both) ranging from 0.654 to 0.765, with sensitivities ranging from 68.1 % to 87.2 %, and specificity from 74.2 % to 80 %). This shows its potential as a valuable biomarker in patients with HCC.

According to a prior analysis, miR-15b-5p had a summary sensitivity and specificity of 60 and 80 %, respectively, for distinguishing liver cirrhosis and persistent HBV and/or HCV from HCC, suggesting its potential use that warrants further investigation [7]. In reference to the extant literature, three studies have explored the possible clinical application of miR-15b-5p. The initial suggestion that miR-15b-5p could be used for the early identification of HCC came from Hung et al. [28]. However, this study did not include explicit diagnostic testing. According to Liu et al.'s study in 2015, binding of miR-130b and miR-15b-5p may enhance the precision of HCC diagnosis by utilizing expression data gathered from HBV carriers, HCC patients, and controls [29]. Chen et al. created three subgroups to assess the clinical potential of miR-15b-5p: patients with cirrhosis, HCC, and healthy individuals. The function of miR-15b-5p was the exclusive focus of the investigations that have been conducted, despite the fact that only two genes, OIP5 and Rab1A, have been identified as direct targets of miR-15b-5p in HCC [30].

The insulin signaling pathway was identified by Kyoto encyclopedia of genes and genomes (KEGG) analysis using bioinformatics analyses. Our investigation revealed that this route was linked to other enriched pathways. Insulin can phosphorylate the insulin receptor substrate (IRS). This phosphorylation enables IRS to interact with PI3K/Akt and MAPK signaling pathways, which are involved in biological processes such as gene transcription, glycogen production, cell glucose consumption, and protein synthesis [31]. Abnormally high levels of IRS-1 and insulin receptors have been linked to carcinogenesis in HCC, which in turn promotes glycogenosis in the precancerous liver and hepatocellular proliferation [[32], [33], [34]]. Tanaka et al. demonstrated that IRS-1 may prevent cell death mediated by transforming growth factor β1, thus increasing the risk of HCC [35]. Researchers have also examined the combined effects of the PI3K/Akt and MAPK signaling pathways, which are thought to be involved in the molecular mechanism of HCC, in addition to IRS-1 [7]. Aconitum coreanum polysaccharide was found to decrease the PI3K/Akt signaling pathway and upregulate the MAPK signaling pathway, thereby inhibiting the expression of pituitary tumor-transforming gene 1, an oncogene, according to Liang et al. [36]. Targeting the PI3K/Akt and MAPK signaling pathways has also been shown to reduce HCC cell growth in a study by Gedaly et al. [37]. Furthermore, it has been discovered that these two signaling pathways independently correlate with HCC cell proliferation [38,39], apoptosis [40,41], migration, invasion and adhesion [[42], [43], [44]]. Adhesion influences HCC carcinogenesis and progression [45,46]. Consequently, we deduced that miR-15b-5p could potentially participate in many pathways related to hepatocellular carcinogenesis. MiR-15b was found by Dai et al. to target hepatocyte nuclear factor 1α, which in turn increased HBV replication [47].

Moreover, additional cancers, including colorectal cancer [48], uterine endometrioid adenocarcinoma [49], and non-small cell lung cancer [50], have also been linked to miR-15b-5p overexpression.

In summary, a high miR-15b-5p level may be one of the likely reasons for HCC carcinogenesis, but it also might be a result of HCC that has already developed, which calls for further research. Practical validation is also necessary to confirm the potential clinical utility of miR-15b-5p, which is expected owing to its notable increase in HCC. Notably, in silico approaches alone have revealed the potential involvement and signaling pathways of miR-15b-5p; hence, additional research is necessary to determine the precise mechanism of miR-15b-5p in HCC.

However, miR-15b has been reported to be upregulated in liver cell faluire (LCF), which should prompt the exclusion of LCF as a cause of elevation of miR-15b [51]. Furthermore, cardiac damage has been shown to induce miR-15b; therefore, cardiac etiologies should be excluded using ECG, echocardiography, and cardiac enzymes before assessing hepatic causes [52].

The treatment of all patients enrolled in this study was not affected at all and all patients received the best management according the standard of care. According to the findings of this study, TLR-4 exhibited 86 % specificity and 85 % sensitivity (AUC:0.885), with a p-value<0.001. However, Elkammah et al. [53] discovered that TLR-4 possessed 62 % sensitivity and 88 % specificity when the cut-off value was greater than 9.91.

Kairaluoma et al. [54] revealed that poor prognosis in HCC can be independently attributed to increased cytoplasmic TLR-4 expression. Additional observations suggest that high levels of TLR-4 nuclear expression could influence prognosis in HCC, such as Shi et al. who stated that TLR-4 contributes significantly to the development of HCC [55].

It is well recognized that chronic inflammatory response is linked to the incidence of liver malignancies. As a key receptor for detecting PAMPs and DAMPs, TLR-4 is involved in both the innate and inflammatory responses. Emerging data indicate that activation of TLR-4 signaling in the liver contributes to the promotion of inflammation- and injury-induced HCC as the primary defense against antigens generated from the gut [56]. According to a recent study, TLR-4 activation drives HCC progression through a positive feedback loop involving COX-2, prostaglandin E2 (PGE2), and STAT3 [57]. TLR-4 expression is positively linked with either IL-6 or CCL2 levels, showing that TLR-4 expression is linked to inflammatory effects, according to prior study results from human HCC samples. Consistent with previously reported findings, it has been demonstrated that TLR-4 signaling activation in HCC confers growth benefits by stimulating cytokine production and proliferation via the Akt and STAT3 pathways. According to their findings, NF-κB-mediated inflammation is triggered by TLR-4 activation, and inflammation is linked to the development of tumors and LIN28 and let-7 miRNAs. While let-7g miRNA and TLR-4 or LIN28A showed a negative association, a positive relationship was observed between TLR-4 and LIN28A. They cultivated PLC5 cells in low-dose LPS-containing media to mimic persistent inflammation with persistent TLR-4 activation; in doing so, they were able to show that TLR-4 and LIN28A mRNA and protein levels increased. Let-7gmiRNA expression decreased as a result of increased LIN28A expression. Moreover, let-7g miRNA targets the 3′-UTR of TLR-4 mRNA, indicating that TLR-4 mRNA might be increased by inhibiting let-7g miRNA [58]. The role of the LIN28/let-7 axis in cancer progression has been previously reported [59]. It has been observed that LIN28-positive tumors exhibit greater aggressiveness in several cancers, such as breast, lung, or prostate cancer, and that let-7 miRNA suppression fosters cancer growth [60]. Hepatitis, cirrhosis, HCC, and virally induced liver damage are among the conditions for which the LIN28/let-7 molecular switch has become a key regulator [61].

Several age-related disorders have been linked to TLR-4 expression. Such disorders may be influenced by their development and progression due to extended exposure to chronic inflammatory reactions caused by TLR-4 hyperactivation or overexpression. Alzheimer's disease, diabetes, emphysema, osteoarthritis, and myocardial diseases are some of these disorders [62], so these conditions must be excluded first.

Although our study yielded important results, it has some potential limitations, such as the relatively small sample size. Furthermore, confounding factors such as other comorbidities or concurrent diseases may impact the accuracy of these diagnostic markers. Finally, factors such as cost-effectiveness, availability, and ease of implementation in different clinical settings should be considered when interpreting results.

More comprehensive research, including HCC linked to different risk factors and different micro-RNA interactions that could be related to HCC, will be helpful. Larger trials are required in the future to confirm these findings.

## 5 Conclusion

The performance of miR-15b-5p in identifying HCC was shown to be positive, outperforming Toll-like receptor4 in detecting HCC patients. As a result, miR-15b-5p has the potential to be a more accurate and predictive biological marker for HCC than Toll-like receptor4. Future research is required to validate these findings, and a comprehensive study including different miRNAs and HCC cases originating from different etiologies is required to better understand the role of miRNAs in this disease and allow for more efficacious diagnosis and prognostic strategies.

## CRediT authorship contribution statement

**Amal Ahmed Mohamed:** Writing – original draft, Methodology, Investigation, Formal analysis, Conceptualization. **Noha Nagah Amer:** Writing – review & editing, Investigation, Formal analysis, Data curation. **Noha Osama:** Writing – review & editing, Visualization, Formal analysis, Conceptualization. **Wael Hafez:** Writing – review & editing, Writing – original draft, Supervision, Methodology, Investigation, Funding acquisition, Formal analysis, Data curation, Conceptualization. **Ali Elsaid Abdelrahman Ali:** Writing – review & editing, Methodology, Formal analysis, Conceptualization. **Mahmoud Maamoun Shaheen:** Writing – review & editing, Resources, Investigation, Data curation. **Ayman Abd Alhady Alkhalegy:** Writing – review & editing, Visualization, Funding acquisition, Data curation. **Eman Alsayed Abouahmed:** Writing – review & editing, Methodology, Formal analysis, Data curation. **Shamel Mohamed Soaida:** Writing – review & editing, Software, Investigation, Data curation. **Lamees A. Samy:** Writing – original draft, Investigation, Formal analysis, Data curation. **Ahmed El-Kassas:** Writing – review & editing, Methodology, Data curation. **Ivan Cherrez-Ojeda:** Writing – review & editing, Validation, Formal analysis, Conceptualization. **Rehab R El-Awady:** Writing – review & editing, Supervision, Project administration, Conceptualization.

## Ethical statement

The tests and investigations were carried out in accordance with the rules and regulations that were applied. Formal informed consent was provided by all patients and controls or by their legal representatives. This study was approved by the Ethics Committee of Al-Azhar University's Faculty of Pharmacy (No. 364).

## Funding

No specific grant from a public, private, or nonprofit funding organization was awarded for this study.

## Declaration of competing interest

The authors declare that they have no known competing financial interests or personal relationships that could have appeared to influence the work reported in this paper.

## References

[1] SEER∗Explorer: An interactive website for SEER cancer statistics [Internet]. Surveillance Research Program, National Cancer Institute; 2024 Apr 17. [updated: 2024 Nov 5; cited 2024 Dec 21]. Available from: https://seer.cancer.gov/statistics-network/explorer/. Data source(s): SEER Incidence Data, November 2023 Submission (1975-2021), SEER 22 registries.

[2] Kocarnik J.M., Compton K., Dean F.E., Fu W., Gaw B.L., Harvey J.D. (2022). Cancer incidence, mortality, years of life lost, years lived with disability, and disability-adjusted life years for 29 cancer groups from 2010 to 2019 A systematic analysis for the global burden of disease study 2019. JAMA Oncol..

[3] Rashed WM, Kandeil MAM, Mahmoud MO, Ezzat S. Hepatocellular Carcinoma (HCC) in Egypt: a comprehensive overview. J. Egypt. Natl. Cancer Inst. 32(1):1–11. 10.1186/s43046-020-0016-x PMC13325438 32372179

[4] Beudeker BJB, Boonstra A. Circulating biomarkers for early detection of hepatocellular carcinoma. Therap Adv Gastroenterol. 13:1756284820931734. 10.1177/1756284820931734 PMC7325534 32647536

[5] Marrero JA, Kulik LM, Sirlin CB, Zhu AX, Finn RS, Abecassis MM, et al. Diagnosis, staging, and management of hepatocellular carcinoma: 2018 Practice guidance by the American association for the study of liver diseases. Hepatology. 68(2):723–750. 10.1002/hep.29913 29624699

[6] Wang H, Peng R, Wang J, Qin Z, Xue L. Circulating microRNAs as potential cancer biomarkers: the advantage and disadvantage. Clin. Epigenet. 10(1). 10.1186/s13148-018-0492-1 PMC5913875 29713393

[7] Pan W.-Y., Zeng J.-H., Wen D.-Y., Wang J.-Y., Wang P.-P., Chen G. (2019 Feb). Oncogenic value of microRNA-15b-5p in hepatocellular carcinoma and a bioinformatics investigation. Oncol Lett [Internet].

[8] Ghafouri-Fard S, Khoshbakht T, Hussen BM, Jamal HH, Taheri M, Hajiesmaeili M. A comprehensive review on function of miR-15b-5p in malignant and non-malignant disorders. Front. Oncol. 12. 10.3389/fonc.2022.870996 PMC9108330 35586497

[9] Yang Y, Feng R, Wang YZ, Sun HW, Zou QM, Li HB. Toll-like receptors: triggers of regulated cell death and promising targets for cancer therapy. Immunol. Lett. 223:1–9. 10.1016/j.imlet.2020.04.002 32311408

[10] Sepehri Z, Kiani Z, Kohan F, Alavian SM, Ghavami S. Toll like receptor 4 and hepatocellular carcinoma; A systematic review. Life Sci. 179:80–87. 10.1016/j.lfs.2017.04.025 28472619

[11] Ghouri YA, Mian I, Rowe JH. Review of hepatocellular carcinoma: epidemiology, etiology, and carcinogenesis. J. Carcinog. 16(1):1–8. 10.4103/jcar.JCar_9_16 PMC5490340 28694740

[12] Goto K, Andres Roca Suarez A, Wrensch F, Baumert TF, Lupberger J. Hepatitis C Virus and Hepatocellular Carcinoma: When the Host Loses Its Grip. Int. J. Mol. Sci. [Internet]. 21(9). Available from: 10.3390/IJMS21093057. PMC7246584 32357520

[13] Llovet JM, Kelley RK, Villanueva A, Singal AG, Pikarsky E, Roayaie S, et al. Hepatocellular carcinoma. Nat. Rev. Dis. Prim. 7(1):1–28. 10.1038/s41572-020-00240-3 PMC13537433 33479224

[14] Axley P, Ahmed Z, Ravi S, Singal AK. Hepatitis C virus and hepatocellular carcinoma: a narrative review. J Clin Transl Hepatol. 6(1):79. 10.14218/JCTH.2017.00067 PMC5863002 29607308

[15] Mohamed AA, Ali-Eldin ZA, El-Serafy M, Ali-Eldin FA, Abdelaziz H. MicroRNAs and clinical implications in hepatocellular carcinoma Prospective Study [Internet]. Available from: 10.4254/wjh.v9.i23.1001. PMC5569275 28878865

[16] Dash S, Aydin Y, Widmer KE, Nayak L. Hepatocellular carcinoma mechanisms associated with chronic HCV infection and the impact of direct-acting antiviral treatment. J. Hepatocell. Carcinoma. 7:45–76. 10.2147/JHC.S221187 PMC7167284 32346535

[17] Mohamed AA, Elsaid OM, Amer EA, Elosaily HH, Sleem MI, Gerges SS, et al. Clinical significance of SNP (rs2596542) in histocompatibility complex class I-related gene A promoter region among hepatitis C virus related hepatocellular carcinoma cases. J. Adv. Res. 8(4):343–349. 10.1016/j.jare.2017.03.004 PMC5388909 28417047

[18] Syed Sameer A, Nissar S. Toll-Like Receptors (TLRs): Structure, Functions, Signaling, and Role of Their Polymorphisms in Colorectal Cancer Susceptibility [Internet]. Available from: 10.1155/2021/1157023. PMC8452412 34552981

[19] Behzadi P, Andrés García-Perdomo H, Karpiński TM. Toll-Like Receptors: General Molecular and Structural Biology [Internet]. Available from: 10.1155/2021/9914854. PMC8181103 34195298

[20] Liu P, Xie S-H, Hu S, Cheng X, Gao T, Zhang C, et al. Age-specific Sex Difference in the Incidence of Hepatocellular Carcinoma in the United States. 10.18632/oncotarget.19245 PMC5620243 28978103

[21] Mcglynn KA, Petrick JL, El-Serag HB. Epidemiology of hepatocellular carcinoma. Rev | Hepatol. 73(S1). 10.1002/hep.31288 PMC7577946 32319693

[22] Dong Yang J, Mohamed EA, Abdel Aziz AO, Shousha HI, Hashem MB, Nabeel MM, et al. Characteristics, management, and outcomes of patients with hepatocellular carcinoma in Africa: a multicountry observational study from the Africa liver cancer consortium and the Africa network for gastrointestinal and liver diseases. Lancet Gastroenterol Hepatol. 2:103–111. 10.1016/S2468-1253(16)30161-3 28403980

[23] Mourad L, El-Ahwany E, Zoheiry M, Abu-Taleb H, Hassan M, Ouf A, et al. Expression analysis of liver-specific circulating microRNAs in HCV-induced hepatocellular Carcinoma in Egyptian patients. Cancer Biol. Ther. 19(5):400–406. 10.1080/15384047.2018.1423922 PMC5915034 29333940

[24] Núñez KG, Sandow T, Patel J, Hibino M, Fort D, Cohen AJ, et al. Hypoalbuminemia is a hepatocellular carcinoma independent risk factor for tumor progression in low-risk bridge to transplant candidates. Cancers. 14(7):1684. 10.3390/cancers14071684 PMC8996921 35406456

[25] Carr BI, Guerra V. Serum albumin levels in relation to tumor parameters in hepatocellular carcinoma patients. Int. J. Biol. Markers. 32(4):391– 396. 10.5301/ijbm.5000300 28862714

[26] Pan WY, Zeng JH, Wen DY, Wang JY, Wang PP, Chen G, et al. Oncogenic value of microRNA-15b-5p in hepatocellular carcinoma and a bioinformatics investigation. Oncol. Lett. 17(2):1695. 10.3892/ol.2018.9748 PMC6341845 30675229

[27] Yi A, Abcd C, Abcdf JC, Liu Y, Shiliang B, Abcdefg L, et al. Plasma miR-15b-5p, miR-338-5p, and miR-764 as Biomarkers for Hepatocellular Carcinoma. Vol. vol. 21. p. 1864–1871. 10.12659/MSM.893082 PMC4497470 26119771

[28] Hung C.H., Hu T.H., Lu S.N., Kuo F.Y., Chen C.H., Wang J.H., Huang C.M., Lee C.M., Lin C.Y., Yen Y.H., Chiu Y.C. (2016). Circulating microRNAs as biomarkers for diagnosis of early hepatocellular carcinoma associated with hepatitis B virus. Int. J. Cancer.

[29] Liu A.M., Yao T.J., Wang W., Wong K.F., Lee N.P., Fan S.T., Poon R.T., Gao C., Luk J.M. (2012). Circulating miR-15b and miR-130b in serum as potential markers for detecting hepatocellular carcinoma: a retrospective cohort study. BMJ Open.

[30] Chen Y., Chen J., Liu Y., Li S., Huang P. (2015). Plasma miR-15b-5p, miR-338-5p, and miR-764 as biomarkers for hepatocellular carcinoma. Med. Sci. Mon. Int. Med. J. Exp. Clin. Res..

[31] Bevan P. (2001). Insulin signalling. J. Cell Sci..

[32] Aleem E., Nehrbass D., Klimek F., Mayer D., Bannasch P. (2011). Upregulation of the insulin receptor and type I insulin-like growth factor receptor are early events in hepatocarcinogenesis. Toxicol. Pathol..

[33] Mohr L., Banerjee K., Kleinschmidt M., Bartolomé Rodriguez M.M., Wands J.R. (2008). Transgenic overexpression of insulin receptor substrate 1 in hepatocytes enhances hepatocellular proliferation in young mice only. Hepatol. Res..

[34] Nehrbass D., Klimek F., Bannasch P. (1998). Overexpression of insulin receptor substrate-1 emerges early in hepatocarcinogenesis and elicits preneoplastic hepatic glycogenosis. Am. J. Pathol..

[35] Tanaka S., Wands J.R. (1996). Insulin receptor substrate 1 overexpression in human hepatocellular carcinoma cells prevents transforming growth factor beta1-induced apoptosis. Cancer Res..

[36] Liang M., Liu J., Ji H., Chen M., Zhao Y., Li S., Zhang X., Li J. (2015). A Aconitum coreanum polysaccharide fraction induces apoptosis of hepatocellular carcinoma (HCC) cells via pituitary tumor transforming gene 1 (PTTG1)-mediated suppression of the P13K/Akt and activation of p38 MAPK signaling pathway and displays antitumor activity in vivo. Tumour Biol.

[37] Gedaly R., Angulo P., Hundley J., Daily M.F., Chen C., Evers B.M. (2012). PKI-587 and sorafenib targeting PI3K/AKT/mTOR and Ras/Raf/MAPK pathways synergistically inhibit HCC cell proliferation. J. Surg. Res..

[38] Wu R., Duan L., Cui F., Cao J., Xiang Y., Tang Y., Zhou L. (2015). S100A9 promotes human hepatocellular carcinoma cell growth and invasion through RAGE-mediated ERK1/2 and p38 MAPK pathways. Exp. Cell Res..

[39] Fang X., Yang D., Luo H., Wu S., Dong W., Xiao J., Yuan S., Ni A., Zhang K.J., Liu X.Y., Chu L. (2017). SNORD126 promotes HCC and CRC cell growth by activating the PI3K-AKT pathway through FGFR2. J. Mol. Cell Biol..

[40] Bao H., Liu P., Jiang K., Zhang X., Xie L., Wang Z., Gong P. (2016). Huaier polysaccharide induces apoptosis in hepatocellular carcinoma cells through p38 MAPK. Oncol. Lett..

[41] Jiang S., Wang Q., Feng M., Li J., Guan Z., An D., Dong M., Peng Y., Kuerban K., Ye L. (2017). C2-ceramide enhances sorafenib-induced caspase-dependent apoptosis via PI3K/AKT/mTOR and Erk signaling pathways in HCC cells. Appl. Microbiol. Biotechnol..

[42] Zhu B., Shi S., Ma Y.G., Fan F., Yao Z.Z. (2012). Lysophosphatidic acid enhances human hepatocellular carcinoma cell migration, invasion and adhesion through P38 MAPK pathway. Hepato-Gastroenterology.

[43] Hsieh Y.H., Hsieh S.C., Lee C.H., Yang S.F., Cheng C.W., Tang M.J., Lin C.L., Lin C.L., Chou R.H. (2015). Targeting EMP3 suppresses proliferation and invasion of hepatocellular carcinoma cells through inactivation of PI3K/Akt pathway. Oncotarget.

[44] Zheng Y., Wang X., Wang H., Yan W., Zhang Q., Chang X. (2014). Bone morphogenetic protein 2 inhibits hepatocellular carcinoma growth and migration through downregulation of the PI3K/AKT pathway. Tumour Biol.

[45] Qiu F.N., Huang Y., Chen D.Y., Li F., Wu Y.A., Wu W.B., Huang X.L. (2016). Eukaryotic elongation factor-1α 2 knockdown inhibits hepatocarcinogenesis by suppressing PI3K/Akt/NF-κB signaling. World J. Gastroenterol..

[46] Zhao J., Dong Q.Z., Zhong F., Cai L.L., Qin Z.Y., Liu Y., Lin C.Z., Qin L.X., He F.C. (2017). NMI promotes hepatocellular carcinoma progression via BDKRB2 and MAPK/ERK pathway. Oncotarget.

[47] Dai X., Zhang W., Zhang H. (2014). Modulation of HBV replication by microRNA-15b through targeting hepatocyte nuclear factor 1α. Nucleic Acids Res..

[48] Li J., Chen Y., Guo X., Zhou L., Jia Z., Tang Y., Lin L., Liu W., Ren C. (2016). Inhibition of miR-15b decreases cell migration and metastasis in colorectal cancer. Tumour Biol.

[49] Wang L., Chen Y.J., Xu K., Xu H., Shen X.Z., Tu R.Q. (2014). Circulating microRNAs as a fingerprint for endometrial endometrioid adenocarcinoma. PLoS One.

[50] Fan L., Qi H., Teng J., Su B., Chen H., Wang C., Xia Q. (2016). Identification of serum miRNAs by nano-quantum dots microarray as diagnostic biomarkers for early detection of non-small cell lung cancer. Tumour Biol.

[51] Yu K., Shi G., Li N. (2015). The function of MicroRNA in hepatitis B virus-related liver diseases: from Dim to Bright. Ann. Hepatol..

[52] Pantaleão L.C., Loche E., Fernandez-Twinn D.S., Dearden L., Córdova-Casanova A., Osmond C., Ozanne S.E. (2024). Programming of cardiac metabolism by miR-15b-5p, a miRNA released in cardiac extracellular vesicles following ischemia-reperfusion injury. Mol. Metabol..

[53] Elkammah M, Gowily A, Okda T, Houssen M. Serum soluble Toll-like receptor 4 and the risk of hepatocellular carcinoma in hepatitis C virus patients. Contemp. Oncol. 24(4):216. 10.5114/wo.2020.102818 PMC7836282 33531868

[54] Kairaluoma V., Kemi N., Huhta H., Pohjanen V.-M., Helminen O. (2021 Apr 3). Prognostic role of TLR4 and TLR2 in hepatocellular carcinoma. Acta Oncol. (Madr.).

[55] Shi L., Zheng X., Fan Y., Yang X., Li A., Qian J. (2019 Dec 24). The contribution of miR-122 to the innate immunity by regulating toll-like receptor 4 in hepatoma cells. BMC Gastroenterol..

[56] Kiziltas S. (2016). Toll-like receptors in pathophysiology of liver diseases. World J. Hepatol..

[57] Lin A., Wang G., Zhao H., Zhang Y., Han Q., Zhang C., Tian Z., Zhang J. (2016). TLR4 signaling promotes a COX-2/PGE2/STAT3 positive feedback loop in hepatocellular carcinoma (HCC) cells. OncoImmunology.

[58] Chen I.T., Cheng A.C., Liu Y.T., Yan C., Cheng Y.C., Chang C.F., Tseng P.H. (2022). Persistent TLR4 activation promotes hepatocellular carcinoma growth through positive feedback regulation by LIN28A/let-7g miRNA. Int. J. Mol. Sci..

[59] Wang T., Wang G., Hao D., Liu X., Wang D., Ning N., Li X. (2015). Aberrant regulation of the LIN28A/LIN28B and let-7 loop in human malignant tumors and its effects on the hallmarks of cancer. Mol. Cancer.

[60] Balzeau J., Menezes M.R., Cao S., Hagan J.P. (2017). The LIN28/let-7 pathway in cancer. Front. Genet..

[61] McDaniel K., Hall C., Sato K., Lairmore T., Marzioni M., Glaser S., Meng F., Alpini G. (2016). Lin28 and let-7: roles and regulation in liver diseases. Am. J. Physiol. Gastrointest. Liver Physiol..

[62] Kim H.J., Kim H., Lee J.H., Hwangbo C. (2023). Toll-like receptor 4 (TLR4): new insight immune and aging. Immun. Ageing : I & A.

